# Left ventricular torsional hysteresis in patients with hypertension: a global parameter for diastolic function

**DOI:** 10.1186/1532-429X-15-S1-O28

**Published:** 2013-01-30

**Authors:** Chun Schiros, Ravi V Desai, Bharath Ambale Venkatesh, Mustafa Ahmed, Shilpi Agarwal, Steven Lloyd, David Calhoun, David McGiffin, Thomas S Denney, Louis J Dell'Italia, Himanshu Gupta

**Affiliations:** 1 Medicine, University of Alabama at Birmingham, Birmingham, AL, USA; 2 Electrical and Computer Engineering, Auburn University, Auburn, AL, USA

## Background

Torsion is an important determinant of left ventricular (LV) systolic and diastolic function. We hypothesized that the area within the torsion volume loop, called the torsional hysteresis (TH), may be an important parameter of diastolic function (DD).

## Methods

60 resistant hypertension (HTN) patients, 40 healthy controls were studied using cardiac MRI with tissue tagging. Volumetric and torsional parameters were evaluated.

## Results

**Figure 1:**
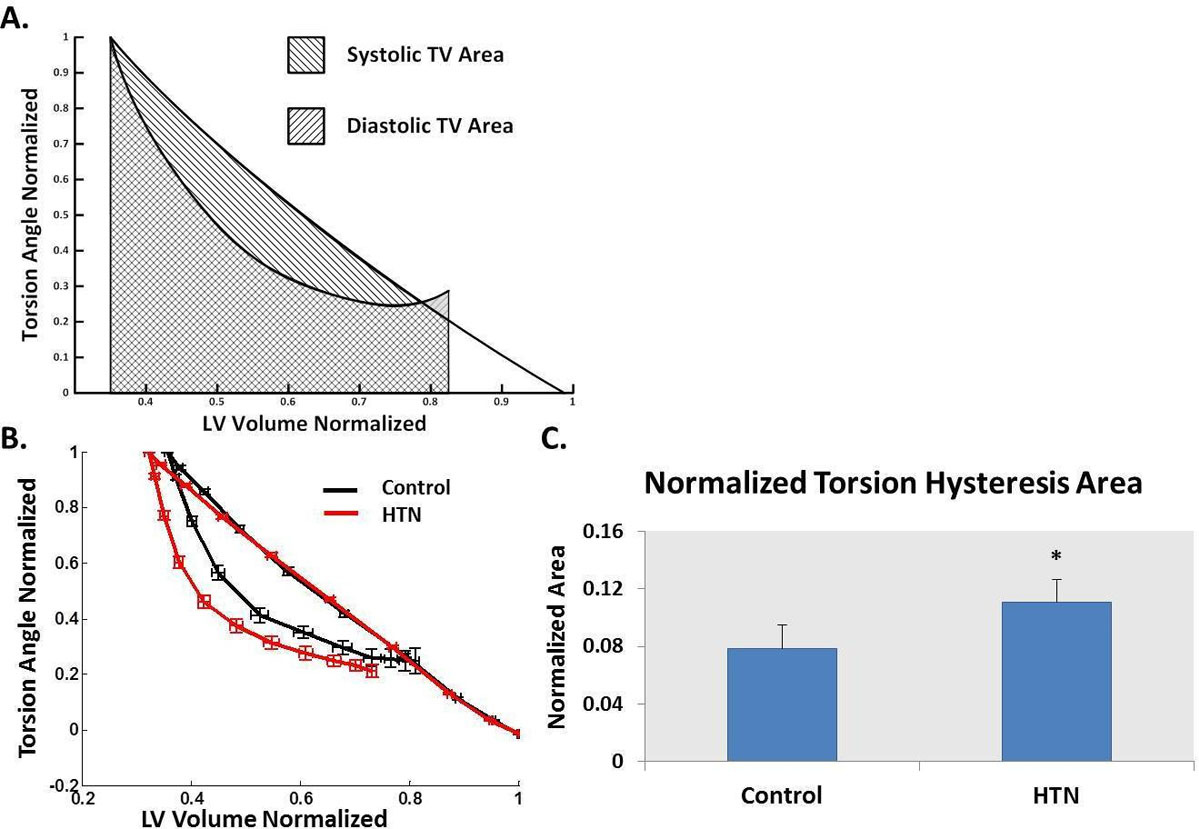
Schematic diagram for calculating torsional hysteresis--the area within the systolic and diastolic arm of the normalized torsion volume (TV) curves (A), normalized TV curves for the two groups (B) and calculated torsional hysteresis (C). Torsion hysteresis is greater in the HTN vs. Control. *, P<0.001 vs. Control; Data points are expressed as mean±standard error.

**Figure 2:**
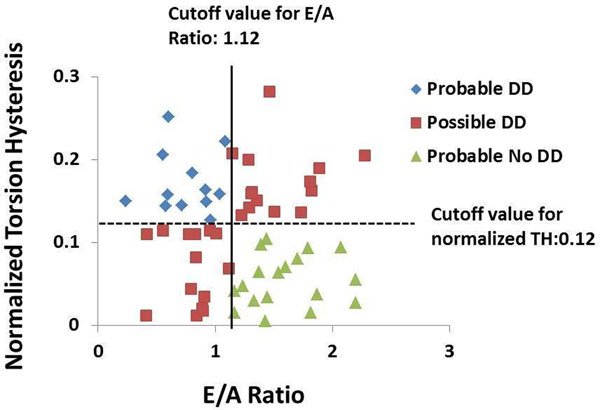
Classification of HTN with and without diastolic dysfunction by E/A ratio and by normalized TH. Probable DD, HTN with DD identified by both E/A ratio and normalized TH; Possible DD , HTN with DD by only one cutoff; Probable No DD, HTN without DD identified by either cutoff; solid black line, cutoff value for E/A ratio; dash black line, cutoff value for normalized TH; DD, diastolic dysfunction; TH, torsional hysteresis.

HTN patients demonstrated concentric remodeling. All HTN patients had preserved ejection fraction (>55%) and normalized peak ejection rate was significantly greater in HTN vs. controls. HTN patients had significantly decreased MRI-measured early filling rate, E/A ratio (1.33±1.13 vs. 2.19±1.07, P<0.0001) and early diastolic mitral annulus velocity normalized to LV length (66.23±20.65 vs. 85.67±29.96 %, P<0.001) vs. controls. Furthermore, the normalized TH was significantly greater in HTN compared controls (0.11± 0.07 vs. 0.079±0.045, P<0.001) (Figure 1). LV normalized TH best correlated with E/A ratio (r=-0.23, P=0.025) but not with LVmass/volume. Mean normalized TH plus 1 standard deviation and mean E/A ratio minus 1 standard deviation of controls were used as cutoffs to identify DD in HTN respectively. Both cutoffs identified ~50% HTN patients with DD (27 by normalized TH, 28 by E/A ratio), among which 13 patients were identified with DD by both cutoffs, as shown in Figure 2.

## Conclusions

TH as measured by area within torsion volume loops was significantly increased in hypertensive concentric remodeling with associated DD. TH took into account not only active and passive recoil processes of the LV diastolic phase but also the systolic phase and represents a heretofore new assessment of diastolic function.

## Funding

NIH-NHLBI P50-HL077100.

